# Modeling X-chromosome inactivation and reactivation during human development

**DOI:** 10.1016/j.gde.2023.102096

**Published:** 2023-08-17

**Authors:** Shafqat A Khan, Thorold W Theunissen

**Affiliations:** Department of Developmental Biology and Center of Regenerative Medicine, Washington University School of Medicine, St. Louis, MO 63110, USA

## Abstract

Stem-cell-based embryo models generate much excitement as they offer a window into an early phase of human development that has remained largely inaccessible to scientific investigation. An important epigenetic phenomenon during early embryogenesis is the epigenetic silencing of one of the two X-chromosomes in female embryos, which ensures an equal output of X-linked gene expression between the sexes. X-chromosome inactivation (XCI) is thought to be established within the first three weeks of human development, although the inactive X-chromosome is reactivated in primordial germ cells (PGCs) that migrate to the embryonic gonads. Here, we summarize our current understanding of X-chromosome dynamics during human development and comment on the potential of recently established stem-cell-based models to reveal the underlying mechanisms.

## Introduction

X-chromosome inactivation (XCI) is an epigenetic process in female placental mammals that equalizes the expression of X-linked genes between the sexes [1]. Achieving dosage compensation during the development of female placental mammals entails dynamic changes between transcriptionally active and inactive X-chromosome states (Xa/Xi). Female murine embryos display imprinted XCI (iXCI) during cleavage stages, whereby the paternal X-chromosome (Xp) is selectively inactivated and remains silenced in extraembryonic lineages [2–4]. The inactive Xp is reactivated in the pluripotent epiblast (EPI) of the mouse blastocyst [5–7]. In contrast, human and cynomolgus monkey pre-implantation embryos exhibit two actively transcribed X chromosomes [8–10]. Upon implantation, either the maternal or paternal X (Xm/Xp) is inactivated through random XCI (rXCI) in humans, mice, and monkeys [8,10,11]. A long noncoding RNA called *X Inactive Specific Transcript (XIST)* is required *in cis* for XCI to occur, as was first demonstrated using mouse embryonic stem cells (ESCs) [12].

Proper XCI is vital for embryonic development. Studies in mice using a paternally transmitted *XIST* deletion demonstrated that biallelic expression of X-linked genes causes embryonic lethality due to malfunctioning extraembryonic tissues [13,14]. The clinical significance of XCI during early human development is underscored by the unexpectedly high prevalence of male babies born from *in vitro fertilization* (IVF) [15,16]. A similar sex ratio distortion in mouse IVF offspring has been attributed to impaired XCI in female embryos [17]. Furthermore, skewed XCI patterns have been linked to infertility, intellectual disability, immune diseases, and cancer [18,19]. However, our knowledge of X-chromosome regulation thus far is mainly based on studies in mice, which — based on a limited number of studies performed on human embryos — appear to have substantially different dosage compensation mechanisms [20–22]. These observations highlight the need for appropriate models of XCI during human development.

The past decade has witnessed remarkable progress in capturing various stages of human embryonic and extraembryonic development using human pluripotent stem cells (hPSCs). Naive hPSCs, which correspond to the preimplantation EPI [23,24], can be reprogrammed into 8-cell embryo-like cells (8CLCs) [25–27] or programmed to acquire a formative state of pluripotency akin to the early postimplantation EPI [28]. In addition, naive hPSCs have a broad potential for extraembryonic differentiation, contributing to both primitive endoderm (PE) and trophectoderm (TE) derivatives [29–33]. In a transformative advance, naive hPSCs have even been shown to self-organize into entire 3D blastocyst-like structures (blastoids) that encompass all three lineages of the preimplantation embryo [34–36]. The rapidly expanding array of stem-cell-based models of human embryogenesis offers an unprecedented opportunity to investigate the mechanisms of XCI *in vitro*.

## Modeling X-chromosome regulation during human preimplantation development

**Figure 1:**
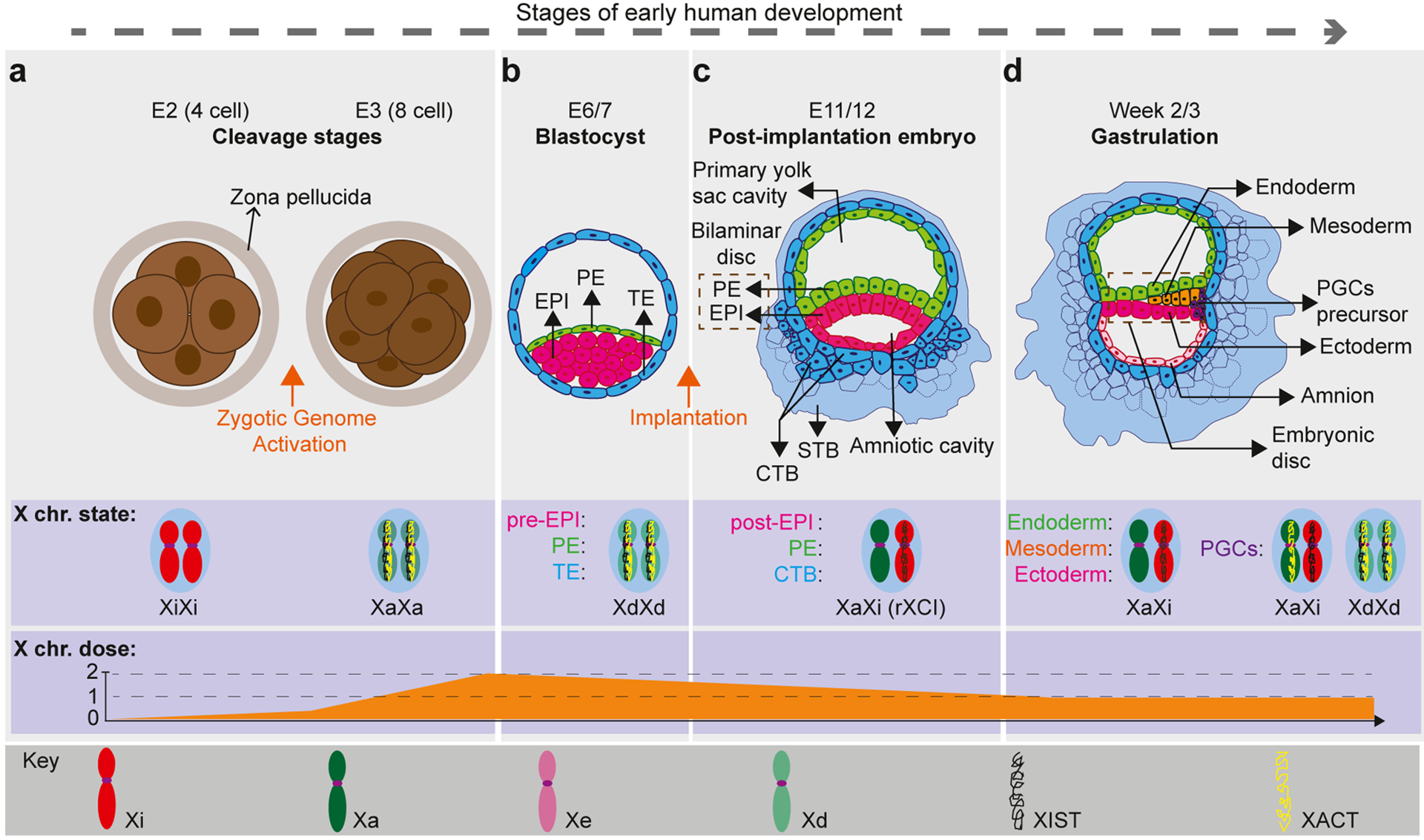
XCI and XCR dynamics during early human development. Cartoon describing the status of *XIST* expression (mono- or biallelic), X-chromosome state (Xa, Xi, Xd: dampened), and X-linked gene expression in major cell lineages observed during early development of female human embryos. **(a)** Shortly after ZGA at E3, both X chromosomes become active with biallelic *XIST* expression. X-linked gene expression increases until E4 and then starts decreasing until it stabilizes during early postimplantation development. **(b)** The totipotent cells of the cleavage embryo give rise to the blastocyst, which contains three cell types: EPI, PE, and TE. The PE and TE contribute to extraembryonic tissues, while EPI contributes to the embryo proper. At this stage, both X chromosomes are in a dampened state where X-linked gene expression decreases, while *XIST* is expressed biallelically. However, the extent of XCD in the early human embryo remains the subject of debate. **(c)** From the implantation of the human embryo at E7 until E14, one of the two X chromosomes is gradually inactivated in all lineages with monoallelic *XIST* expression from the Xi. Inactivation of one of the X chromosomes takes place independently in each cell where either the maternal or paternal X is randomly inactivated (rXCI), which is clonally maintained thereafter. **(d)** By the end of week 3, the human embryo forms three germ layers (endoderm, mesoderm, and ectoderm). During this time, PGCs, the precursors of the gametes, develop from the posterior EPI or early amnion and migrate to the developing gonads via the hindgut. At this stage, the XCI pattern established in the early postimplantation EPI is maintained in all three germ layers: monoallelic *XIST* expression with one Xi. However, the Xi is reactivated in PGCs through the process of XCR. This is thought to entail a gradual process that starts with the specification of PGCs, continues while the PGCs migrate through the hindgut, and is completed after the PGCs complete their migration to the gonads. In the end, XCR in PGCs results in an X-chromosome state very similar to that of preimplantation EPI cells: biallelic *XIST* expression with dampened X-linked gene expression (XdXd).

The divergence in dosage compensation mechanisms between mice and humans is best exemplified during preimplantation development. Female mouse embryos display iXCI, in which the Xp is inactivated and marked by upregulation of *XIST* (Xa^*XIST*–^Xi^*XIST*+^), shortly after zygotic genome activation (ZGA) [6]. In contrast, RNA fluorescence *in situ* hybridization (FISH) and single-cell RNA sequencing (scRNA-seq) indicate that both X chromosomes are actively transcribed in human pre-implantation embryos [8,9,37]. Lanner and colleagues reported that X-linked gene expression first increases from embryonic day E3 to E4 and then gradually decreases until E7 in all three lineages of the blastocyst (EPI, PE, and TE) [9] (Figure 1a–b). These observations led to the proposition that dosage compensation in human preimplantation embryos is achieved by lowering gene expression from both X chromosomes, a process named X-chromosome dampening (XCD). Surprisingly, *XIST* is expressed by both actively transcribed X chromosomes in human preimplantation embryos (Xd^*XIST*+^Xd^*XIST*+^) [8,9,37]. This provides another important distinction with the mouse embryo in which *XIST* is downregulated in the preimplantation EPI [5–7].

A subsequent study reanalyzed XCI dynamics in human preimplantation embryos using a different bioinformatic pipeline and reported a decrease in biallelic and a concomitant increase in monoallelic X-linked gene expression, suggestive of rXCI, rather than dampening, in the human blastocyst [38]. However, analyzing an independent scRNA-seq dataset of human embryos, Tang and colleagues concluded that the majority of single cells in E6 blastocysts still express X-linked genes in a balanced manner from both the maternal and paternal alleles [11]. Regardless of the exact time when rXCI is completed, it is evident from these studies that human embryos are subject to unique dosage compensation mechanisms that cannot be adequately modeled in mice, such as the lack of iXCI and the uncoupling of *XIST* expression from XCI.

**Figure 2:**
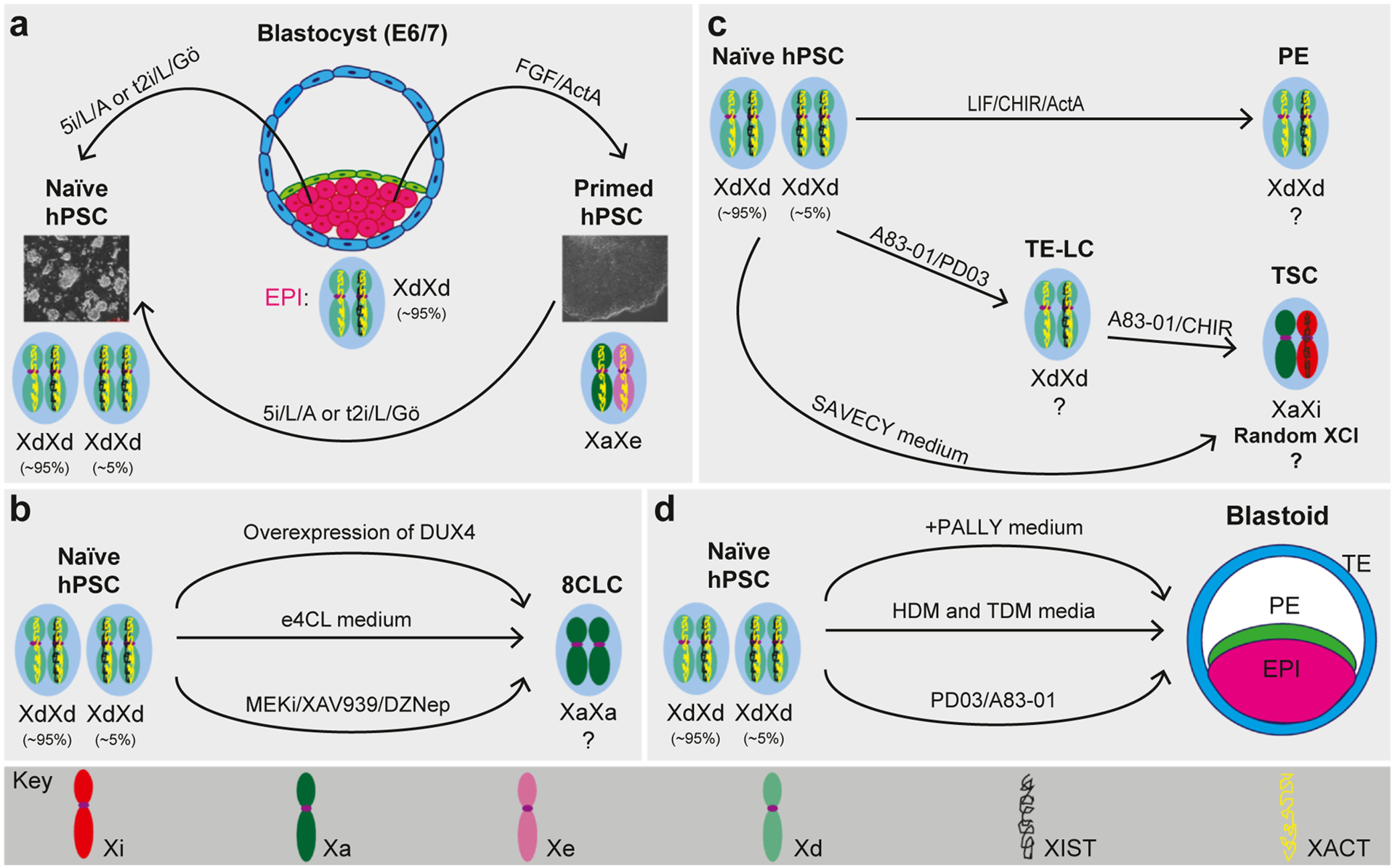
Stem-cell models of X-chromosome regulation during human preimplantation development. Schematic representation of embryonic and extraembryonic cell identities during human preimplantation development that can be accessed from naive hPSCs to model X-chromosome regulation. **(a)** hPSCs representing discrete pluripotent states can be isolated from human blastocysts under specific culture conditions: 5i/L/A and t2i/L/Gö capture naive hPSCs that correspond to preimplantation EPI, while basic FGF (bFGF) and activin capture primed hPSCs that correspond to postimplantation EPI. Female-primed hPSCs exhibit an X-chromosome state similar to postimplantation EPI cells: one Xi with monoallelic *XIST* expression. However, upon prolonged culture, primed hPSCs show X-chromosome erosion, which is characterized by loss of *XIST* expression and partial reactivation of the Xi. In contrast, female naive hPSCs exhibit two actively transcribed X chromosomes and XCD (XdXd), similar to preimplantation EPI cells. However, the extent of mono- or biallelic *XIST* expression in naive hPSCs varies between culture conditions and derivation methods (primed-to-naive reversion vs. *de novo* derivation from human blastocysts). **(b)** Methods to generate 8CLCs from naive hPSCs as a basis for modeling X-chromosome dynamics at the very beginning of human development. Naive hPSCs can be directly reprogrammed into 8CLCs in e4CL media (middle arrow) [27]. 8CLCs can also be enriched by overexpressing DUX4 in naive hPSCs (upper arrow) [25] or by culture in the presence of MEKi/XAV939/DZNep/p53 activator/PARP1i [26]. However, these enriched 8CLCs are transient in nature and so far cannot be stably maintained long-term. The X-chromosome status of 8CLCs remains to be investigated. **(c)** Accessing human extraembryonic lineages from naive hPSCs and expected XCI states. Naive hPSCs can be directly differentiated into PE-like cells in the presence of leukemia inhibitory factor (LIF), the GSK3 inhibitor CHIR99021 (CHIR), and recombinant activin A [50]. Naive hPSCs can also acquire a trophoblast identity by direct application of hTSC media containing SB431542, A83–01, valproic acid, epidermal growth factor, and Y-27632 (SAVECY) [29,31,32] or sequential induction of a transient trophectoderm-like cell (TE-LC) state using PD03/A83–01 ± JAKi/BMP4, and subsequent progression to a postimplantation cytotrophoblast (CTB) identity using either SAVECY or A83–01, CHIR99021, and EGF (ACE) [30,33]. Owing to their preimplantation identity, female PE and TE-LCs are expected to show an X-chromosome state similar to naive hPSCs: two dampened X chromosomes (XdXd) with mono- or biallelic *XIST* expression. In contrast, hTSCs represent a postimplantation identity and, thus, are expected to show random X-chromosome inactivation (rXCI) with monoallelic *XIST* expression. **(d)** 3D blastocyst-like structures (blastoids) generated from naive hPSCs enable the simultaneous assessment of XCI dynamics in embryonic (EPI) and extraembryonic (PE and TE) lineages. Thus far, blastoids have been generated from 5i/L/A naive hPSCs using hypoblast differentiation medium (HDM) and trophoblast differentiation medium (TDM) (middle arrow) [35], and naive hPSCs reprogrammed in medium containing PD0325901, XAV939, Gö 6983, and LIF (PXGL) using either medium containing PD0325901, A83–01, LPA, hLIF, and Y-27632 (PALLY) (top arrow) [34] or a combination of PD03/A83–01/Y27632 small molecules (bottom arrow) [36].

Conventional or ‘primed’ hPSCs resemble the post-implantation EPI on the cusp of gastrulation [39,40], a stage of human development when XCI is close to completion [11]. Consequently, most female primed hPSCs have already initiated XCI, although a subset of primed hPSC lines harbor an Xi that becomes partially reactivated (‘eroded’) with prolonged culture (XaXe) [41–43] (Figure 2a, *right*). This eroded XCI status has been linked to poor differentiation behavior and hampers disease modeling for X-linked mutations [44]. Recently developed protocols for inducing naive pluripotency have enabled the generation of female hPSCs in which a majority of cells exhibit X-chromosome reactivation (XCR) [45–47]. Encouragingly, naive hPSCs derived using one of two conditions, t2i/L/Gö [23] or 5i/L/A [24], also express *XIST* from the actively transcribed X-chromosome [37,45] (Figure 2a, *left*). However, most naive hPSCs show monoallelic expression of *XIST* and only a minority of cells (5–30%) fully mirror the Xd^*XIST*+^Xd^*XIST*+^ pattern seen in the human blastocyst. This fraction varies depending on the naive culture medium and derivation method (primed-to-naive resetting vs. *de novo* derivation from the blastocyst). Furthermore, Plath and colleagues reported a downregulation of X-linked genes as cells transition from an *XIST*-negative XaXa intermediate state to the *XIST*-positive XaXa naive state [45]. These data suggest that naive hPSCs are subject to XCD, as previously reported in human embryos [9], and that this process may be dependent on *XIST*. Further studies are required to clarify whether XCD affects only one X-chromosome in naive hPSCs with monoallelic *XIST* and both X chromosomes in cells with biallelic *XIST*. Thus, while currently available naive hPSCs do not capture all facets of X- chromosome regulation in the early human embryo, they provide a starting point for mechanistic studies into human-specific forms of dosage compensation.

How can naive hPSCs help us unravel the mechanisms of X-chromosome regulation during human preimplantation development? First, the role of *XIST* in XCD can be further investigated by comparing the levels of X-linked gene expression from the *XIST*-expressing X-chromosome to that of the *XIST*-negative X-chromosome at the single-cell level [45]. More direct evidence about the role of *XIST* could be obtained by deleting one or both of its copies in naive hPSCs and measuring the impact on X-linked gene expression. Second, Rougeulle and colleagues reported that the hominid-specific lncRNA *X Active Coating Transcript (XACT)* co-accumulates, yet barely overlaps, with *XIST* on actively transcribed X chromosomes in both human pre-implantation embryos and naive hPSCs [37]. These observations raised the suspicion that *XACT* might compete with *XIST* accumulation *in cis*. This hypothesis can be explored further by genetically perturbing *XACT* in naive cells and determining the impact on *XIST* localization and X-linked gene expression. It is worth pointing out, however, that *XACT* seems to be dispensable for maintaining Xi erosion, since its deletion does not alter the expression of X-linked gene in female-primed hPSCs [48]. Third, it will be of interest to determine whether histone modifications participate in achieving dosage compensation in the human preimplantation embryo. Elsässer and colleagues recently reported that inhibition of the polycomb subunit EZH2 does not cause global upregulation of X-linked gene expression in naive cells [49], excluding an active role for H3K27me3 in XCD. Finally, naive hPSCs provide a unique model system for investigating the impact of 3D conformational changes along the X chromosome. A recent study reported that naive hPSCs derived in 5i/L/A display a shift towards more Xa-like folding conformations, but surprisingly this did not correlate with *XIST* expression state [80].

The above studies need not be restricted to naive hPSCs under self-renewing conditions. By reverting naive hPSCs into an 8-cell embryo-like state [25–27], it is now possible to establish an *in vitro* model system for dosage compensation mechanisms at an even earlier stage of human development when *XIST* is first induced [38] (Figure 2b). In addition, by applying culture conditions that support the differentiation of naive hPSCs into PE [50] or TE [30,33], we can model human-specific dosage compensation mechanisms in extraembryonic lineages of the preimplantation embryo (Figure 2c). Finally, the generation of blastoids from naive hPSCs [34–36] offers an integrated embryo model in which the functional significance of candidate regulators of XCI, such as *XIST* and *XACT*, on all three blastocyst lineages can be interrogated simultaneously (Figure 2d).

## Modeling random X-chromosome inactivation during human postimplantation development

Early postimplantation development is the executive phase of XCI when a random choice is made to silence either the Xm or Xp, which is then clonally propagated through cell division. Currently, our only direct insights into the kinetics of rXCI during human embryogenesis come from the study by Tang and colleagues, who performed scRNA-seq analysis of human embryos cultured *in vitro* up to E12 [11]. They concluded that allele-specific expression of X-linked genes in female embryos gradually accumulates between E8 and E12 in the EPI, PE, and TE lineages (Figure 1c). In addition, they reported that the expression ratio of X-linked and auto-somal genes (X:A ratio) in male embryos was close to 1. This observation is consistent with Ohno’s hypothesis that genes located on the single X-chromosome in male cells are upregulated by nearly twofold to compensate for dosage differences with autosomes, a process named X-chromosome upregulation [51]. On the other hand, the X:A ratio in female embryos was above 1, suggesting that rXCI had been initiated but not completed by E12.

Saitou and colleagues recently performed a detailed analysis of the dosage compensation program in cynomolgus monkey embryos [10]. They noted the formation of an intermediate XCI state in female embryos in which both X chromosomes are marked by repressive histone modifications and a compacted chromatin structure, but nonetheless remain actively transcribed. This active intermediate XCI state was already present in the TE around implantation, but emerged more slowly in other postimplantation lineages [10], suggesting that XCI may be accelerated in the primate TE. It remains unclear why the *XIST*-coated Xa in human preimplantation embryos does not accumulate repressive histone modifications in a similar manner, but the authors speculate that this difference may be attributed to hominid-specific expression of *XACT* [37].

**Figure 3:**
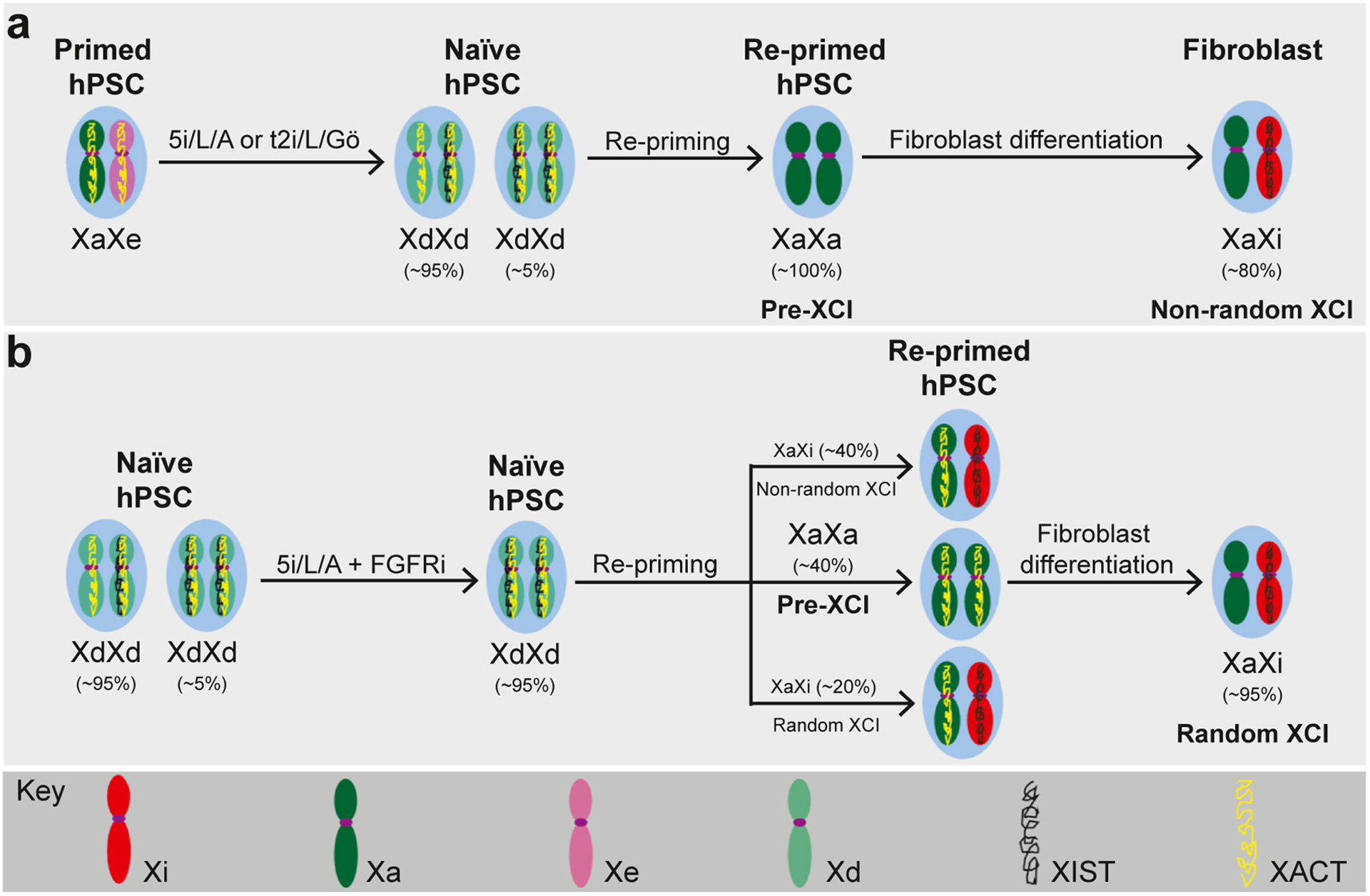
Stem-cell models of XCI during differentiation of naive hPSCs. Schematic representation of two reports describing XCI dynamics during differentiation of naive hPSCs. Naive hPSCs are not directly responsive to embryonic lineage-inductive cues, but can complete germ layer differentiation after transition into a postimplantation EPI identity by repriming or capacitation. **(a)** Sahakyan et al. reported that repriming of naive hPSCs derived in 5i/L/A or t2i/L/Gö generated a cell state with two active X chromosomes (XaXa) without *XIST* expression, while further differentiation into fibroblasts resulted in nonrandom XCI (non-rXCI) and monoallelic *XIST* expression [45]. **(b)** An et al. reported that the addition of an FGF receptor inhibitor to 5i/L/A naive hPSCs enhances the fraction of naive cells with biallelic *XIST* expression and enables rXCI upon repriming and subsequent fibroblast differentiation [52].

Can we use human stem cells to model the initiation of rXCI? Currently available naive hPSCs initiate *de novo* XCI upon differentiation, but inactivation is biased toward the X-chromosome that was silenced in the parental primed cells [45,46] (Figure 3a). Wang and colleagues reported that overcoming autocrine FGF signaling enhances the fraction of naive cells with biallelic *XIST* expression and facilitates rXCI upon differentiation [52] (Figure 3b). However, supplementing the 5i/L/A cocktail with an FGF receptor inhibitor was associated with reduced cell survival and did not support induction of naive pluripotency. We recently showed using a dual-color fluorescent reporter system that naive hPSCs initiate rXCI at the locus of a gene called *Mutations in methyl CpG binding protein 2 (MECP2)* upon differentiation into human trophoblast stem cells (hTSCs) [53]. These experiments were performed using an alternative naive induction cocktail containing PD0325901, XAV939, GDC-0994, Gö 6983, Y-27632, and Activin A (PXGGY/A), which also promotes biallelic *MECP2* reporter activity in naive cells [54]. Hence, hTSC derivation from naive hPSCs may provide a tractable system for studying the initiation of rXCI during early human development. It will be important to define the extent of rXCI at multiple X-linked genes, the relative kinetics of XCI during embryonic and extraembryonic differentiation, and the influence of specific naive culture conditions on the XCI process.

A question of particular interest is to identify regulators of rXCI during the differentiation of naive hPSCs. The *XIST antisense RNA transcript* (*Tsix)* is a well-known repressor of *XIST* on the Xa in mice [55], but its locus is poorly conserved in humans, and human *TSIX* appears to colocalize with *XIST* on the Xi [56]. Studies in mouse ESCs have also identified several putative *XIST* activators on the future Xi, such as *Just proximal to XIST (Jpx)* and *Ring finger protein 12* (*Rnf12)* [57,58]. Rougeulle and colleagues recently reported that inhibition of *JPX* reduces the expression of *XIST* in primed hPSCs [59]. Therefore, it will be instructive to genetically perturb candidates such as *JPX* or *RNF12* in naive hPSCs and determine whether these factors contribute to the initiation of rXCI upon differentiation. Ongoing efforts to culture human blastoids through implantation stages may soon allow investigation of rXCI in multiple embryonic and extraembryonic lineages simultaneously.

## Modeling X-chromosome reactivation in the human germline

**Figure 4:**
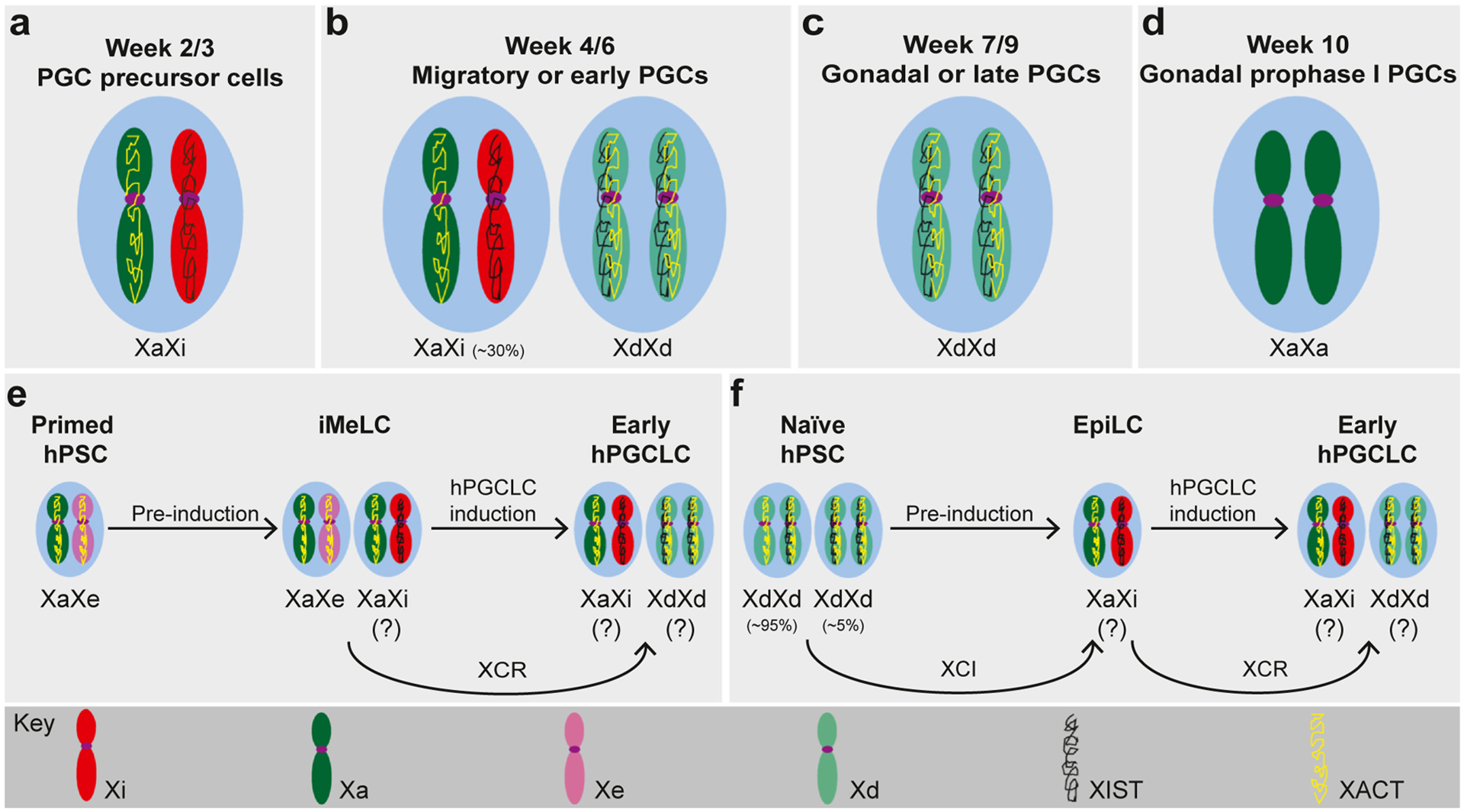
Stem-cell models of XCR in the human germline. Schematic representation of XCR *in vivo* **(a–d)** and available model systems **(e–f)** to interrogate the human germline *in vitro* by generating hPGCLCs from various hPSC states. **(a)** During weeks 2–3 of development, cells from the posterior EPI or early amnion get specified as hPGC precursor cells, and in females, they show the same XCI status as postimplantation EPI [62]. **(b)** During weeks 4–6, hPGCs begin their migration from the EPI to the gonads through the hindgut and continue to develop. At this stage, female hPGCs show a heterogeneous X-chromosome state: the majority of hPGCs show complete XCR, while about 30% hPGCs remain in full or partial XCI state. The XCR state of hPGCs is marked by XCD together with biallelic expression of *XIST*, *XACT*, and other X-linked genes, similar to preimplantation EPI [63,64,66,69,70]. **(c)** By week 9, hPGCs reach the gonads and, in females, start differentiating into oogonia. Gonadal hPGCs show a homogeneous XCD state with biallelic expression of *XIST*, *XACT*, and other X-linked genes [63,64,71]. **(d)** At week 10 of development, female hPGCs enter into meiosis. Before entering into meiosis, hPGCs silence both *XIST* and *XACT* and upregulate X-linked gene expression, thereby changing X-chromosome state from XCD to XCR [63,64,71]. **(e)** The most-established model of hPGCLC induction starts from primed hPSCs and passes through an intermediate cell state called incipient mesoderm-like cells (iMeLCs). However, the eroded state of the X-chromosome is known to be maintained during differentiation and may not support XCR during germ cell induction [73]. **(f)** Naive hPSCs have been used to generate hPGCLCs through an intermediate state called epiblast-like cells (EpiLCs). This *in vitro* method offers an excellent opportunity to study sequential cycles of rXCI (preimplantation to postimplantation stage) and XCR (postimplantation to germ cell development stage) observed during early development of female embryos [79].

Although XCI is completed during early postimplantation development, studies in mice, cynomolgus monkeys, and humans have shown that the Xi becomes reactivated in female primordial germ cells (PGCs), the precursors of the gametes [60,61]. During the first 2–3 weeks of development, human primordial germ cells (hPGCs) are specified from the posterior EPI or early amnion [62] (Figure 1d). They migrate to the embryonic gonads during weeks 4–5 where they differentiate into oogonia or gonocytes in the ovaries and testes [63,64]. During week 10, oogonia and gonocytes begin their sexual differentiation: in females, oogonia differentiate into oocytes through meiotic prophase, while gonocytes differentiate into fetal spermato-gonia in males [63,64]. These stages of hPGC development are accompanied by extensive epigenetic reprogramming, including DNA demethylation, imprint erasure, and XCR in females (Figure 4a–d) [65–67].

Payer and colleagues investigated X-chromosome dynamics in detail during mouse germ cell development *in vitro* and reported that XCI is crucial for proper germ cell differentiation, while XCR is necessary for meiotic progression [68]. These findings highlight the importance of timely XCI and XCR for germ cell maturation and suggest a potential link between X-chromosome dosage compensation and developmental progression in germ cells. Several studies concluded that XCR has taken place in human PGCs (hPGCs) by the fourth week of development based on biallelic expression of X-linked genes and an absence of H3K27me3 domains [66,69]. However, Chouva de Sousa Lopes and colleagues reported that roughly 30% of female hPGCs still exhibit incomplete or ongoing X reactivation between weeks 4 and 9 of development (Figure 4b) [70]. More recently, Clark and colleagues demonstrated using RNA FISH that female hPGCs harbor two actively transcribed X chromosomes by week 7 (Figure 4c) [71]. Interestingly, these actively transcribed X chromosomes were also coated by *XACT* and *XIST*, mimicking the X-chromosome profile seen in preimplantation EPI [8,9,37]. Clark and colleagues also noted a reduced X:A ratio in female hPGCs, indicating that the female germline is subject to XCD (Figure 4b–c). This Xd^*XIST*+^Xd^*XIST*+^ state lasts until hPGCs enter into meiosis during week 10, at which point female germ cells silence both *XIST* and *XACT* and upregulate X-linked gene expression (Figure 4d).

While the abovementioned studies have illuminated our understanding of the X-chromosome status in hPGCs starting around 4 weeks of development, it still remains unclear how XCR is initiated in hPGCs during earlier stages. Some insights can be obtained from studies in closely related mammals. A recent study in pigs provided evidence for *XIST* repression and extensive XCR in PGCs even before they migrate to the embryonic gonads [72]. Similarly, Saitou and colleagues noted an initial wave of *XIST* repression during PGC specification in female cynomolgus monkey embryos from E13 to E17, which coincided with an increase in biallelic X-linked gene expression [10]. This was followed by the disappearance of H3K27me3-enriched domains between E22 and E28 and re-expression of *XIST* at later stages. Based on these studies, we hypothesize that XCR in nascent hPGCs likely involves an initial wave of *XIST* repression concomitant with a switch to biallelic X-linked gene expression.

Can we model the initiation of XCR in the human germline *in vitro*? Several protocols have been developed to model human gametogenesis *in vitro* (Figure 4e–f). Primed hPSCs can be differentiated into hPGC-like cells (hPGCLCs) that express markers such as SOX17, TFAP2C, and BLIMP1, thus corresponding to weeks 2–4 of an early or premigratory hPGC phenotype (Figure 4e) [73,74]. Excitingly, Saitou and colleagues were even able to induce the differentiation of hPGCLCs into early meiotic oogonia-like cells in xenogeneic-reconstituted ovaries with mouse embryonic ovarian somatic cells [75,76]. These oogonia-like cells, but not the parental hPGCLCs, showed partial (~20%) XCR without *XIST* expression. However, since erosion of the Xi in primed hPSCs is maintained during differentiation [42,71], primed cells do not provide an ideal starting point to dissect XCI and XCR dynamics in the human germline (Figure 4e). A potential solution is to prevent erosion of XCI in primed hPSCs, which has been linked to WNT signaling [77]. In fact, Saitou and colleagues recently reported that WNT inhibition prevents erosion of XCI in primed cynomolgus monkey PSCs, providing a better model system of XCI dynamics during germ cell differentiation [78]. As an alternative approach, Reik and colleagues have shown that naive hPSCs can acquire germ cell fate in response to similar cues as mouse ESCs (Figure 4f) [79]. It will be of interest to determine whether hPGCLC induction from naive hPSCs involves successive waves of XCI and XCR, as expected based on studies in animal models (Figure 4f). The establishment of a cellular model that captures X-chromosome dynamics in the human germline will be of tremendous value given the difficulties in investigating weeks 2–4 of human development. Finally, the extended culture of integrated embryo models, such as blastoids, may offer a complementary model system to elucidate XCI dynamics during germ cell induction, provided that conditions can be established that support their development through gastrulation stages.

## Discussion

Our understanding of dosage compensation has come a long way since Mary Lyon first proposed that one of the two X chromosomes in female cells is randomly inactivated to balance the dosage of X-linked gene expression between the sexes [1]. However, most studies on X inactivation have been performed in the mouse system, which — as we have learned from the handful of studies in human embryos — only partially recapitulates the human dosage compensation program. Given ethical and practical restrictions on manipulating human embryos, stem-cell-based models of early human development offer an unprecedented opportunity to define human-specific dosage compensation mechanisms. It is important to bear in mind that the X-chromosome status of stem-cell-based embryo models is inextricably linked to that of the stem cells from which they originate. If XCI is already established, as in most conventional (primed) hPSCs, the embryo models will likely maintain XCI, as has been demonstrated during *in vitro* differentiation [42]. In contrast, naive hPSCs display two active X chromosomes and are capable of undergoing *de novo* XCI upon differentiation [37,45,46]. Nevertheless, some important differences with the human embryo remain, such as the lack of biallelic *XIST* coating in the entire cell population and biased XCI during differentiation, requiring further refinement of naive culture media. Coupled with the rapid pace of stem-cell-based embryo modeling, we anticipate that it will soon be possible to reconstitute the complete dynamics of XCI and XCR from the 8-cell embryo-like stage to hPGCLC specification *in vitro*. These approaches will allow investigation of several important questions that have remained elusive thus far, such as the role of the lncRNAs *XACT* and *XIST* in XCD, the regulation or rXCI during differentiation, and the events leading to XCR in the human germline.

## Data Availability

No data were used for the research described in the article.

## Key abbreviations

XCD: Xchromosome dampening
XCI: X-chromosome inactivation
iXCI: imprinted XCI
rXCI: random XCI
XCR: X-chromosome reactivation
Xa: active X-chromosome
Xd: dampened X-chromosome
Xe: eroded X-chromosome
Xi: inactive X-chromosome
Xm: maternal X-chromosome
Xp: paternal X-chromosome
